# Plasma levels of soluble VEGF receptor isoforms, circulating pterins and VEGF system SNPs as prognostic biomarkers in patients with acute coronary syndromes

**DOI:** 10.1186/s12872-018-0894-1

**Published:** 2018-08-15

**Authors:** Edward C. A. Marks, Tom M. Wilkinson, Chris M. Frampton, Lorraine Skelton, Anna P. Pilbrow, Tim G. Yandle, Chris J. Pemberton, Robert N. Doughty, Gillian A. Whalley, Chris J. Ellis, Richard W. Troughton, Maurice C. Owen, Neil R. Pattinson, Vicky A. Cameron, A. Mark Richards, Steven P. Gieseg, Barry R. Palmer

**Affiliations:** 1 Christchurch Heart institute, Department of Medicine, University of Otago, PO Box 4345, Christchurch, New Zealand; 2 Department of Medicine, Faculty of Medicine and Health Sciences, University of Auckland, Auckland, New Zealand; 3 Department of Medicine, Dunedin School of Medicine, University of Otago, Auckland, New Zealand; 4 School of Biological Sciences, University of Canterbury, Christchurch, New Zealand; 5 Canterbury Scientific Ltd, 71 Whiteleigh Ave, Christchurch, New Zealand; 6 Cardiovascular Research Institute, National University of Singapore, Singapore, Singapore; 7 School of Health Sciences, College of Health, Massey University Wellington, Wellington, New Zealand

**Keywords:** sFlt-1, KDR, Neopterin, Acute coronary syndromes, Mortality, Prognosis

## Abstract

**Background:**

Development of collateral circulation in coronary artery disease is cardio-protective. A key process in forming new blood vessels is attraction to occluded arteries of monocytes with their subsequent activation as macrophages. In patients from a prospectively recruited post-acute coronary syndromes cohort we investigated the prognostic performance of three products of activated macrophages, soluble vascular endothelial growth factor (VEGF) receptors (sFlt-1 and sKDR) and pterins, alongside genetic variants in VEGF receptor genes, *VEGFR-1* and *VEGFR-2.*

**Methods:**

Baseline levels of sFlt-1 (VEGFR1), sKDR (VEGFR2) and pterins were measured in plasma samples from subgroups (*n* = 513; 211; 144, respectively) of the Coronary Disease Cohort Study (CDCS, *n* = 2067). DNA samples from the cohort were genotyped for polymorphisms from the *VEGFR*-1 gene SNPs (rs748252 *n* = 2027, rs9513070 *n* = 2048) and *VEGFR*-2 gene SNPs (rs2071559 *n* = 2050, rs2305948 *n* = 2066, rs1870377 *n* = 2042).

**Results:**

At baseline, levels of sFlt-1 were significantly correlated with age, alcohol consumption, NTproBNP, BNP and other covariates relevant to cardiovascular pathophysiology. Total neopterin levels were associated with alcohol consumption at baseline. 7,8 dihydroneopterin was associated with BMI. The A allele of *VEGFR*-2 variant rs1870377 was associated with higher plasma sFlt-1 and lower levels of sKDR at baseline. Baseline plasma sFlt-1 was univariately associated with all cause mortality with (*p* < 0.001) and in a Cox’s proportional hazards regression model sFlt-1 and pterins were both associated with mortality independent of established predictors (*p* < 0.027).

**Conclusions:**

sFlt-1 and pterins may have potential as prognostic biomarkers in acute coronary syndromes patients. Genetic markers from VEGF system genes warrant further investigation as markers of levels of VEGF system components in these patients.

**Trial registration:**

Australian New Zealand Clinical Trials Registry. ACTRN12605000431628. 16 September 2005, Retrospectively registered.

**Electronic supplementary material:**

The online version of this article (10.1186/s12872-018-0894-1) contains supplementary material, which is available to authorized users.

## Background

Vascular endothelial growth factor (VEGF) signalling is a critical step in angiogenesis [1]. VEGF-A is one of five related growth factors, which bind primarily to three tyrosine kinase receptors with different affinity. The structurally related VEGF receptors, denoted VEGFR1/FMS-like tyrosine kinase-1 (Flt-1), VEGFR2/Kinase insert domain receptor (KDR) and VEGFR3 (Flt-4) have overlapping, but distinct expression patterns, with Flt-1 present in monocytes, macrophages and vascular endothelial cells [2, 3], KDR in vascular endothelial cells and Flt-4 in lymphatic endothelial cells. The complexity of the expression profile of these receptors is increased further by alternative splicing [2].

Cardiac ischaemia modulates the regulation and expression of several VEGF family ligands and receptors via Hypoxia Inducible Factor-1 (HIF-1) [4]. Flt-1 is also upregulated by hypoxia through a HIF-1-dependent mechanism [5] and appears to play an important role in monocyte activation [6], likely targeting phospholipase C and phosphoinositide 3-kinase, but its kinase-inducing activity is poor [2, 7, 8]. Although the exact mechanism of Flt-1 function is still under debate, there is strong evidence for VEGF-A binding to KDR to promote the growth of vascular endothelial cells inducing a potent angiogenic response to hypoxic conditions. This results in the remodelling of arteries and production of new blood vessels and vasodilation [9] to relieve ischaemic stress in CVD [10].

Soluble Flt-1 (sFlt-1) is an alternatively spliced, circulating form of Flt-1 with affinity for VEGF equal to that of the membrane bound isoform. However, in contrast to Flt-1, sFlt-1 is produced mainly by endothelial cells and deposited in their extracellular matrix [11]. Acting as a decoy, sFlt-1 (also up-regulated by hypoxia) binds circulating VEGF, inhibiting the angiogenic action of VEGF binding to membrane bound KDR on arterial endothelium [12]. Consequently sFlt-1 has been implicated as a negative regulator of angiogenesis. Elevated levels of sFlt-1, following acute myocardial ischemia in coronary artery disease patients, have been associated with increased mortality in post-MI patients [13].

Well-developed coronary collateral arteries are associated with improved survival in patients with coronary artery disease [14]. Complementary to the VEGF system driving angiogenesis, a key factor in forming new blood vessels is the attraction of monocytes and their activation as macrophages [15]. 7,8 Dihydroneopterin (7,8NP) and neopterin, produced by activated macrophages, are known markers of immune activation [16]. Recent studies have shown that neopterin levels are higher in patients with acute MI, as opposed to healthy subjects [17, 18]. Atherosclerotic plaques collected during carotid endarterectomy show varying levels of both 7,8 NP and neopterin [19]. In addition, a significant correlation between serum neopterin and complex coronary artery stenosis has been found [20], indicating the potential of neopterin as a prognostic tool in CVD.

Since sFlt-1, sKDR and pterins may all be products of activated macrophages following cardiac ischemia, we investigated the prognostic performance of these markers shortly after an acute coronary syndrome (ACS) event in a well-characterised cohort, the Coronary Disease Cohort Study [21–27]. As SNPs from VEGFR-2 have previously been shown to be associated with the development of CVD [28], VEGF receptor gene variants were also evaluated as prognostic markers.

## Methods

### Coronary disease cohort study

The Coronary Disease Cohort Study (CDCS) recruited 2067 patients after admission to Christchurch or Auckland City Hospitals with a diagnosis of ACS, from July 2002 to January 2009. Inclusion criteria included ischemic discomfort plus one or more of ECG changes (ST-segment depression or elevation of ≥0.5 mm, T-wave inversion of ≥3 mm in ≥3 leads, or left bundle branch block), elevated levels of cardiac markers, a history of coronary disease, age of ≥65 years, and a history of diabetes or vascular disease [24]. Patients with serious co-morbidity that reduced their life expectancy to < 3 years (e.g. end-stage renal failure, cancer) were excluded from the study. Recruitment included a wide spectrum of age, both sexes and patients with established risk factors for CHD such as hypertension and diabetes. Plasma was collected at a baseline clinic, a median of 32 days following index admission for ACS. Demographic and clinical data was collected at baseline including blood pressure, ECG, echocardiography, family and personal medical history, height, weight, and medication prescribed. Plasma samples were assayed for natriuretic peptides and other analytes. Patients were followed for a median of 5.04 years. Patients attended follow-up clinics 3–5 months and 12–14 months post-onset of ACS, and participants completed questionnaires at two and three years post-discharge. Ethnicity was self-declared and categorized as Maori/Pacific Islander, European, Other or Unknown. Standardized transthoracic echocardiography was performed at baseline and at each follow-up clinic using a GE Vivid 3 (GE Medical Systems) ultrasound system at Christchurch Hospital and Philips ATL HDI 5000 and ie33 (Philips Healthcare, Bothell, WA) ultrasound systems at the University of Auckland as described previously [21, 23]. The study was approved by the New Zealand (NZ) Multi-Region Ethics Committee and all participating patients provided written, informed consent.

### Clinical events

Clinical events were determined from recruitment questionnaires, planned follow-up clinic visits, consultation of patient notes, the NZ Ministry of Health and hospital Patient Management System databases, linked through the National Health Index (NHI) number for each patient. Survival times were calculated from the date of index admission. The investigation conforms to the principles outlined in the Declaration of Helsinki and Title 45, U.S. Code of Federal Regulations, Part 46.

### Evaluation of angiographic data

In the Christchurch subgroup of the cohort collateral vessels were graded according to the Rentrop classification: 0: no filling of any collateral vessels, 1: filling of side branches of the artery to be perfused by collateral vessels without visualization of the epicardial segment; 2: partial filling of the epicardial artery by collateral vessels; and 3: complete filling of the epicardial artery by collateral vessels [29], while blinded to other clinical data. Coronary artery anatomy, severity of coronary stenoses and the myocardium at risk were assessed according to the Brandt score [30] in the same subgroup.

### Analyte measurements

Plasma samples were collected and stored at − 80°C. sFlt-1 and sKDR were analysed using chemiluminescent quantitative sandwich enzyme immunoassays (R&D Systems, Minneapolis) detection limits, sFlt-1 3.5 pg/mL and sKDR 4.6 pg/mL. Samples from patients recruited earliest at Christchurch Hospital, for which sufficient plasma was available, were chosen for assay of sFlt-1, sKDR and pterins to maximise length of follow-up and therefore accumulated numbers of clinical events and the strength of survival analyses. Circulating levels of BNP and N-terminal proBNP (NT-proBNP) were assayed as previously described [31]. HPLC measurement of pterins was performed using a Shimadzu, Kyoto, Japan, Sil-20A HPLC with auto-sampler, RF-20Axls fluorescence detector. A 10 μL plasma sample was injected onto a Luna 5 μm SCX 100 Å, 250 × 4.6 mm column with a mobile phase of 100% 20 mM ammonium phosphate (pH 2.5) pumped at 1 mL/min as previously described [32]. Neopterin was detected by fluorescence at wavelength emission of 438 nm and excitation of 353 nm, whereas 7,8 NP was detected following oxidation to neopterin by iodide. All analysis of plasma analytes was conducted in duplicate.

### DNA extraction and SNP genotyping

Extraction of genomic DNA for genotyping was performed as described previously [23]. DNA samples were genotyped for the rs2071559 (T-604C), rs2305948 (G1192A), rs1870377 (T1719A) polymorphisms in the VEGFR-2 receptor gene (encoding KDR) and rs748252 (C8764T) and rs9513070 (A189427G) in the VEGFR-1 receptor gene (encoding Flt-1). The SNPs from VEGFR-1 were chosen with reference to dbSNP and International HapMap Project data, as at the time of initiating this study there were no prior reports of genetic association studies of polymorphisms in VEGFR-1 and CVD. Genotyping was performed by real-time PCR, using allele-specific TaqMan genotyping probes (Applied Biosystems) in 5 μL reaction volumes in 384-well plates, including 1× Roche LightCycler 480 Probes Master mix and 100 ng of genomic DNA in a Roche LC480 (Roche Diagnostics, Auckland).

### Statistical analysis

Univariate analyses to test for associations between SNP genotype and demographic, analyte levels and echocardiographic measurements were performed using χ^2^ and ANOVA tests. Skewed data were log-transformed before analysis and geometric means with 95% confidence intervals reported and adjusted for age, and the time between index admission and baseline sampling. The survival of stratified groups was compared using Kaplan-Meier analysis and the log-rank test. Independent associations between genotype and survival were tested using Cox proportional hazards multivariate analysis including the following established predictors; age, gender, previous MI, Type 2 diabetes, baseline creatinine, physical activity and NTproBNP levels as previously justified [23, 31]. Multivariate linear regression models were based on covariates showing univariate association with sFlt-1. The study had power to detect a hazard ratio (HR) of > 1.7 as statistically significant (two tailed α < 0.05, 90% power) in the CDCS cohort for analysis of those patients assayed for sFlt-1. Levels of sFlt-1 at baseline were assayed in baseline plasma samples from 513 patients selected from the CDCS cohort. Samples from patients with a baseline plasma sample available at the earliest recruitment dates were chosen to maximise number of events on follow up for inclusion in survival analyses (recruited between July 2002 and August 2007). Levels of KDR were assayed in baseline plasma samples from 211 patients, in whom sFlt-1 levels were available (recruited between July 2002 and February 2004). Levels of pterins were assayed in 144 baseline plasma samples from patients in whom sFlt-1 levels were available (recruited between September 2006 and May 2007). In the genetic association part of the study there was 80% power to detect HRs > 1.4 with rare homozygote groups having a frequency of 20% of total cohort. An additive genetic model was used unless stated otherwise. All analyses were performed using SPSS version 22 (IBM, Armonk, USA). Statistical significance was set at the 5% level (*p* < 0.05).

## Results

### VEGFR SNP genotypes and CDCS cohort data

Baseline features of the CDCS cohort are summarized in Table 1. Genotypes were obtained from DNA samples from patients from the cohort for the *VEGFR*-1 gene SNPs (C8764T rs748252 [*n* = 2027], A189427G rs9513070 [*n* = 2048]) and for the *VEGFR*-2 gene SNPs (T-604C rs2071559 [*n* = 2050], G1192A rs2305948 [*n* = 2066], T1719A rs1870377 [*n* = 2042]) as shown in Table 2. The genotype distributions conformed to the Hardy-Weinberg equilibrium (*p* ≥ 0.998).

**Table 1:** Baseline characteristics of the CDCS cohort

Baseline characteristics	n	Mean ± SE or n (%)
Male Gender	2067	1483 (71.7%)
Index event diagnosis:		
Unstable Angina	2067	553 (26.8%)
ST-elevation MI	2067	460 (22.2%)
Non-ST-elevation MI	2067	1054 (51.0%)
Age at baseline (years)	2067	66.6 ± 0.27
Ethnicity (European, Maori & Pasifika, Other, Unknown)	2067	85.9,4.8,3.0,6.3%
Previous MI	2053	619 (30.2%)
Antecedent Hypertension	2050	1069 (52.1%)
Type II diabetes	2061	336 (16.3%)
Renal disease	2052	205 (10.0%)
BMI (kg/m^2^)	2039	27.6 ± 0.11
Tobacco Use (never smoked)	2067	750 (36.3%)
Alcohol Use (non-drinker)	2064	516 (25.0%)
LVEF	1967	57.3% ± 0.27
Discharge Medications		
ACE inhibitor	2067	1178(57.0%)
β-blocker	2067	1778 (86.0%)
Diuretic	2067	566 (27.4%)
Statin	2067	1826 (88.3%)

**Table 2:** Genotype frequencies of polymorphisms investigated in this study in the CDCS cohort

	n	AA^a^, n (%)	Aa^a^, n (%)	aa^a^, n (%)
rs748252 (*VEGFR1*)	2027	811 (40.0%)	931 (45.9%)	285 (14.1%)
rs9513070 (*VEGFR1*)	2048	811 (39.6%)	918 (44.8%)	319 (15.6%)
rs1870377 (*VEGFR2*)	2042	1146 (56.1%)	760 (37.2%)	136 (6.7%)
rs2071559 (*VEGFR2*)	2050	516 (25.2%)	1050 (51.2%)	484 (23.6%)
rs2305948 (*VEGFR2*)	2066	1665 (80.6%)	381 (18.4%)	20 (1.0%)

^a^A = major allele, a = minor allele (rs748252 A = C, a = T; rs9513070 A = A, a = G; rs1870377 A = T, a = A; rs2071559 A = C, a = T; rs2305948 A = G, a = A)

### Analyte measurements

Levels of sFlt-1 at baseline were assayed in baseline plasma samples from 513 patients selected from the CDCS cohort. Baseline characteristics of this group are summarised and compared to the remainder of the cohort in Additional file 1: Table S1. Patients with a baseline plasma sample available from the earliest recruitment dates (between July 2002 and August 2007) were chosen to maximise the number of events during follow up for inclusion in survival analyses. Baseline levels of sFlt-1 had a geometric mean of 105 pg/mL (95% CI 102–110 pg/mL, CV = 52.8%) and were weakly correlated with age (*n* = 513, *r* = 0.167, *p* < 0.001), troponin I (*n* = 509, *r* = 0.267, *p* < 0.001), NTproBNP (*n* = 513, *r* = 0.216, *p* < 0.001) and BNP levels (n = 513, *r* = 0.181, *p* = 0.003) at baseline. A weak inverse correlation of sFlt-1 with LVEF (*n* = 498, *r* = − 0.144, *p* < 0.001) was observed. Levels of sFlt-1 differed significantly (*p* = 0.035) between patients who were current drinkers of alcohol (*n* = 306, mean = 106 pg/mL) and non-drinkers (*n* = 143, mean = 101 pg/mL). Using a multivariate linear regression model, age, troponin I, and time to plasma sampling (all *p* < 0.001) were independently associated with sFlt-1 levels.

Levels of KDR, assayed in baseline plasma samples from 211 patients, in whom sFlt-1 levels were available, had a geometric mean of 10,500 pg/mL (95% CI 10200–10,900 pg/mL, CV = 24.1%) at baseline, with an inverse correlation with sFlt-1 (*n* = 185, *r* = − 0.184, *p* = 0.027) and age (*n* = 211, *r* = − 0.153, *p* < 0.037). KDR was significantly higher (*p* = 0.025) in drinkers (Current Drinkers: *n* = 135, mean = 11,200 ± 221 pg/mL; Ex-/Non-drinkers: *n* = 76, mean 10,300 ± 295 pg/mL). As preliminary analysis provided no indication that KDR was associated with any parameter of clinical interest, the decision was made to assay no further samples for KDR.

**Fig. 1:**
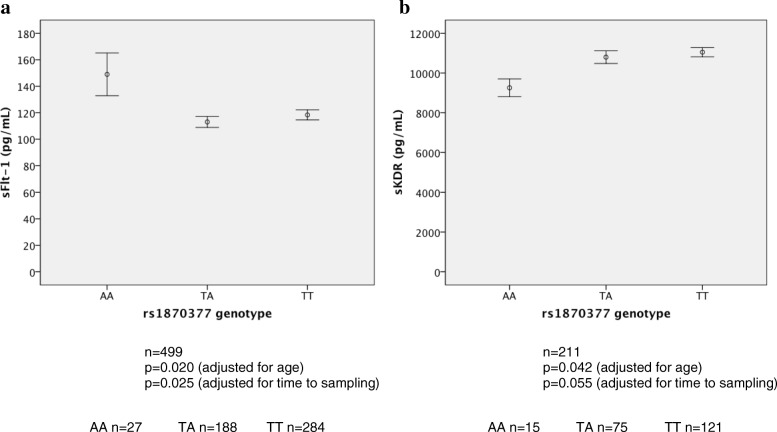
Plots of the relationship between rs1870377 genotype and (**a**) baseline sFlt-1, (**b**) baseline sKDR

Investigation of the association of VEGFR receptor gene SNPs with baseline characteristics revealed that the genotype of the*VEGFR*-2 SNP rs1870377 A allele was significantly associated with higher levels of sFlt-1 and lower levels of sKDR at baseline (Table 3, Fig. 1). These associations did not appear to be influenced by any differences in allele frequencies between European and non-European ethnic groups (Additional file 1: Table S2). There were no significant associations between the other SNPs assayed and either sFlt-1 or KDR levels.

**Table 3:** Genetic associations with VEGFR levels in baseline plasma and angiogram measurements

**a)** ***VEGFR2*** - **rs1870377**							
	**n**	TT	n	TA	n	AA	***p***
Age (years)	1141	66.5 ± 0.37	758	70.0 ± 0.43	136	66.3 ± 1.02	0.646
Male Gender (F/M)	1141	823 (72.1%)	758	530 (69.9%)	136	106 (77.9%)	0.143
sFlt-1 (pg/ml)	284	106(100–112)	188	104(96.8–111)	27	132(110–157)	0.044
sKDR (pg/ml)	121	11,049 ± 235	75	10,799 ± 299	15	9255 ± 668	0.038
Brandt Score	580	3.26 ± 0.13	384	3.39 ± 0.16	69	3.70 ± 0.45	0.519
**b)** ***VEGFR1*** - **rs748252**							
	**n**	CC	n	CT	n	TT	***p***
Age (years)	807	66.9 ± 43	929	66.6 ± 0.40	284	66.3 ± 0.75	0.771
Male Gender Gender	807	595 (73.7%)	929	656 (70.6%)	284	195 (68.7%)	0.178
sFlt-1 (pg/ml)	222	124 ± 4.50	224	112 ± 3.80	50	119 ± 9.06	0.277
sKDR (pg/ml)	90	11,100 ± 307	77	10,500 ± 277	18	10,300 ± 643	0.290
Brandt Score	427	3.19 ± 0.15	457	3.35 ± 0.15	135	3.86 ± 0.30	0.054

Levels of pterins (neopterin, *n* = 144; 7,8 NP, *n* = 135; total pterins, *n* = 138) were assayed in baseline plasma samples from among patients in whom sFlt-1 levels were available. Baseline characteristics of this group are summarised and compared to the remainder of the cohort in Additional file 1: Table S3. Levels of total pterins ranged between 16.58 nM and 624.6 nM, mean of 94.4 ± 9.1 nM and a standard deviation of 107.08 nM. Total pterins trended higher in females (78.5 [62.7–98.4] nM) than males (60.1 [50.9–70.8] nM)(*p* = 0.058); and lower in current drinkers of alcohol (*n* = 138, drinkers 56.8 [49.1–65.7], ex−/non-drinkers 78.0 [62.0–98.1] nM, *p* = 0.020), but the latter association was lost after adjustment for patient age and gender. 7,8 NP levels had a geometric mean of 37.7 (31.4–45.3) nM, and neopterin 17.8 (15.9–20.0) nM. Neopterin was weakly positively correlated with NTproBNP (*n* = 143, *r* = − 0.167, *p* = 0.034).

### Clinical outcome in the CDCS cohort

**Fig. 2:**
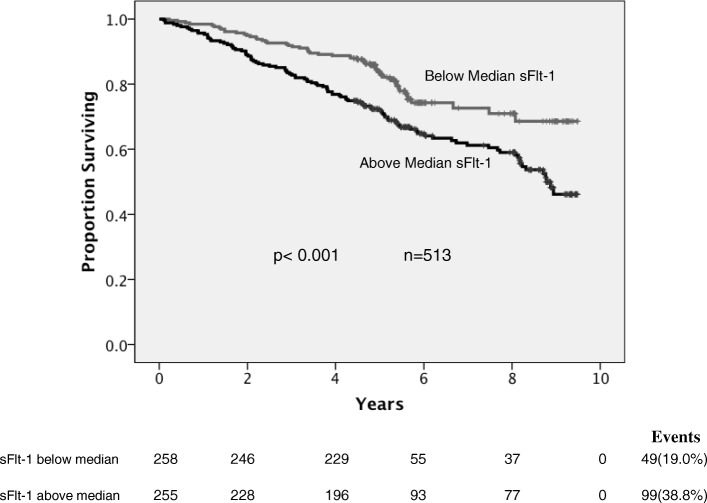
Kaplan-Meier survival analysis of the CDCS cohort stratified by above and below median sFlt-1 levels

Baseline levels of plasma sFlt-1 were associated with all-cause mortality (*p* < 0.001) (Fig. 2). In multivariate survival analysis, sFlt-1 predicted mortality independent of age, gender, NT-proBNP, creatinine, troponin I, physical activity score, alcohol consumption, history of previous MI, diabetes, ethnicity, ACS diagnosis and time to sampling (*p* = 0.023) (Table 4). This was also true for the endpoint of deaths attributable to cardiovascular (CV) causes (CV deaths *n* = 87, above median sFlt-1 mortality = 21.6%, below median sFlt-1 mortality = 12.6% *p* = 0.029). ROC analysis is also presented in Additional file 2: Figure S1 comparing sFlt-1 and NTproBNP as predictors of mortality at 5 years of follow-up. The VEGFR2 SNP rs1870377 was associated with reduced risk of HF readmission (AA v TA/TT, *n* = 2033, events = 379, hazard ratio = 0.74, 95% CI 0.58–0.95, *p* = 0.019, adjusted for age, time to sampling, gender, and ethnicity).

**Table 4:** Cox’s proportional hazards regression model for mortality in the subgroup of the CDCS cohort assayed for sFlt-1 (*n* = 476, 143 deaths)

	Coefficient	SE	Wald	df	Significance	Hazard Ratio	95% CI for HR	
							Lower	Upper
Age at baseline	0.07	0.01	35.1	1	< 0.001	1.07	1.05	1.10
Male Gender	0.14	0.22	0.004	1	0.951	1.01	0.66	1.56
Log_10_ NTpro-BNP at baseline^a^	0.90	0.29	9.71	1	0.002	2.46	1.40	4.33
Log_10_ sFlt-1 at baseline^a^	1.10	0.49	5.15	1	0.023	3.00	1.16	7.76
Creatinine baseline	0.01	0.001	16.8	1	< 0.001	1.01	1.00	1.01
Log_10_Troponin I at baseline^a^	0.02	0.19	0.02	1	0.900	1.02	0.70	1.50
Physical Activity (scale 1–4)^b^	−0.32	0.07	18.5	1	< 0.001	0.73	0.63	0.84
Alcohol consumption category^c^			3.25	2	0.197			
Non-drinker v Current Drinker	−0.21	0.12	2.99	1	0.84	0.81	0.63	1.03
Non-drinker v Ex-drinker	0.08	0.17	0.25	1	0.62	1.09	0.79	1.50
Previous Myocardial Infarction	0.49	0.20	6.17	1	0.013	1.62	1.11	2.38
Type 2 Diabetes	0.32	0.22	2.08	1	0.149	1.38	0.89	2.14
Ethnicity			1.05	3	0.789			
European v Maori/Pasifika	0.24	0.60	0.16	1	0.691	1.27	0.39	2.11
European v Other	0.98	1.05	0.88	1	0.349	2.67	0.34	20.8
European v Unknown	11.5	201	0.003	1	0.954	< 0.01	< 0.01	2.9 × 10^166^
Acute Coronary Syndrome Diagnosis			1.31	2	0.518			
NSTEMI v STEMI	−0.20	0.28	0.48	1	0.488	0.82	0.47	1.43
NSTEMI v Unstable Angina	−0.21	0.21	1.01	1	0.315	0.81	0.54	1.22
Time to Sampling^d^	−0.001	0.01	0.02	1	0.901	0.99	0.98	1.02

^a^Hazard Ratio represents the change in risk for every 10-fold increase in analyte level

^b^Score of 1 = sedentary, 2 = < 30 min activity on > 2 days/week, 3=≥30 min on 2 days/week, 4= ≥30 min on ≥3 days/week

^c^Current Drinker, Ex-drinker or Non-drinker

^d^Days between index admission and plasma sampling at recruitment visit

**Fig. 3:**
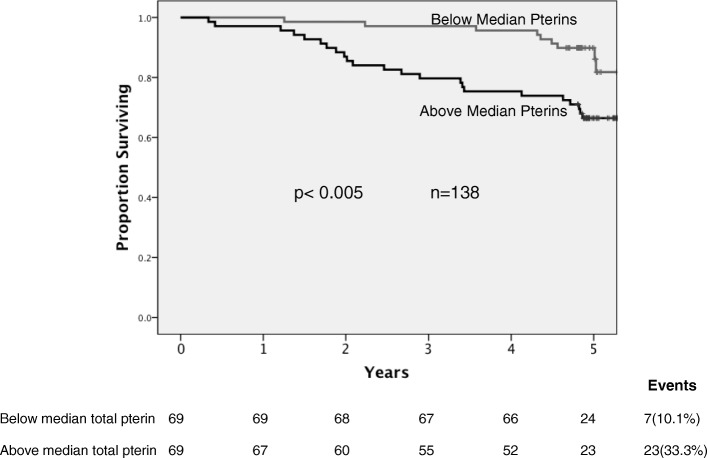
Kaplan-Meier survival analysis of the CDCS cohort stratified by above and below median total-pterin levels

All-cause mortality (*n* = 138) was greater for those with above-median compared with below-median total pterin levels (Fig. 3.; 33.3% versus 10.1%, respectively; *p* = 0.005) and greater for patients with above-median 7,8 NP (28.2% versus 15.3%, *p* = 0.026). In a Cox’s proportional hazards model total pterin levels were predictive of death independent of age, time to sampling, sFlt-1 and NT-proBNP levels (Table 5).

**Table 5:** Cox’s proportional hazards regression model for mortality in the subgroup of the CDCS cohort assayed for pterins (*n* = 138, 32 deaths)

	Coefficient	SE	Wald	df	Significance	Hazard Ratio	95% CI for HR	
							Lower	Upper
Age at baseline	0.06	0.02	7.20	1	0.007	1.06	1.02	1.10
Log_10_ NTpro-BNP at baseline^a^	1.28	0.53	5.87	1	0.015	3.59	1.28	10.1
Log_10_s-Flt-1 at baseline^a^	3.06	1.38	4.87	1	0.027	21.2	1.41	319
Log_10_Total pterins at baseline^a^	2.16	0.54	16.3	1	< 0.001	8.70	3.04	24.9
Time to sampling^b^	0.004	0.02	0.04	1	0.847	1.00	0.97	1.05

^a^Hazard Ratio represents the change in risk for every 10-fold increase in analyte level

^b^Days between index hospital admission and plasma sampling

### Angiogram measurements

Levels of sFlt-1 at baseline were not significantly correlated with Brandt score (sFlt-1 n=337, *r* = 0.078 *p* = 0.156; sKDR *n* = 124, *r* = -0.201, *p* = 0.026). BNP and NT-proBNP levels at baseline were associated with Brandt Score (BNP *n* = 1068, *r* = 0.206, *p* < 0.001; NTproBNP *n* = 1068, *r* = 0.222, *p* < 0.001). The VEGFR-1 SNP rs748252 TT genotype group trended towards higher age-adjusted Brandt score than other genotype groups (Table 3) and this appeared to be consistent across ethnic groups (Additional file 1: Table S2). Rentrop classification for extent of angiographically apparent coronary collateral vessels was not significantly associated with levels of soluble VEGF receptor, pterins or any of the five SNPs genotyped for this study.

## Discussion

Higher levels of sFlt-1, total pterins, and 7,8 NP were all univariately associated with mortality in the CDCS cohort, whereas, higher levels of neopterin and sKDR were not. In multivariate analysis total pterin, and sFlt-1 were independent markers of mortality post-acute coronary event. sFlt-1 was not more accurate as a predictor of mortality than the established biomarker of CHD NTproBNP, as indicated by the ROC analysis in Additional file 2: Figure S1, but may be complementary to existing predictors of outcome.

In a similar study of patients with coronary artery disease undergoing angiography, sFlt-1 was not found to be a significant independent predictor (*p* = 0.125) of adverse outcomes (hazard ratio = 0.57) [33]. Contrary to this, we found sFlt-1 was an independent predictor of all-cause mortality (*p* < 0.023) and CVD death over a median of 5.04 years follow-up after an index acute coronary event. A possible explanation for the difference between our findings and those of Matsumoto et al. [33] is the more severe coronary disease in the CDCS cohort, as levels of upregulated sFlt-1 may correlate with ischaemic burden. Since the risk of adverse events is higher in patients with elevated sFlt-1 (above 117.3 pg/ml), this level may have clinical utility in risk stratification and in guidance for surveillance and intensity of management. It is likely that continuous activation of intramural inflammation plays a role in the occurrence of cardiovascular events.

The positive association of sFlt-1 and level of alcohol consumption is interesting and has been reported elsewhere [34]. The U-shaped association of alcohol with CVD [35], may in part be explained by its association with sFlt-1 levels in the plasma of acute coronary syndrome patients, potentially linking the consumption of alcohol with angiogenesis via alcohol’s alternative activation of the VEGFR, KDR. Levels of sFlt-1 and sKDR were not associated with angiographic measurements in a robust fashion. The VEGFR SNPs assayed were also not strongly associated with measurements of collateral formation or myocardium at risk. While this suggests that none of these markers have value as predictors of collateral angiogenesis, the weak associations detected suggest a thorough investigation of these metrics and a more exhaustive set of genetic markers is warranted. Linear regression analysis suggests that as well as age, troponin I levels were associated with sFlt-1 levels at baseline. It may be that levels of myocardial damage influence expression of sFlt-1 in post-acute coronary patients.

Levels of total pterins and 7,8 NP, were significantly associated with all-cause mortality, in agreement with past research relating inflammation to risk of death after acute CVD events [36]. Neopterin has been previously presented as an independent marker of acute coronary events and all-cause mortality after an initial event [37]. However, that prior report only measured neopterin and the finer-grained measurements presented here bring additional information pertinent to prognosis. Enhanced T-cell activity, resulting in increased production of interferon-γ, is implicated in the pathogenesis of CVD [38, 39]. Total pterin production by monocytes and macrophages, is primarily a response to stimulation by interferon-γ released by activated T-lymphocytes [40]. Therefore, the levels of total pterin observed may reflect activation of cell-mediated immunity, in turn related to disease severity and hence to mortality post-CVD event.

None of the gene polymorphisms investigated were associated with mortality, despite the fact that the *VEGFR2* SNP rs1870377 AA genotype was associated with higher levels of sFlt-1 and lower levels of sKDR. rs1870377 genotype has been associated with response to drug treatment and survival in cancer, femoral head osteonecrosis and recurrent pregnancy loss (www.snpedia.com/index.php/rs1870377), suggesting a link to angiogenic potential. Three polymorphisms of KDR have been shown to be associated with risk of CHD in Han Chinese [28]. A microsatellite in *FLT-1* (*VEGFR1*) has been studied in relation to coronary artery lesions in Japanese Kawasaki disease patients, but was not associated with CAD [41]. Furthermore, mouse model studies have shown that KDR knockout is not compatible with vascular development [42]. Our findings are at best suggestive, needing further validation in extensive surveys of genetic links between angiogenesis, angiogenic biomarkers and clinical outcome in cohorts of CHD patients.

Limitations of the study include: 1) missing data for some parameters limited the power of this study to explore their association with genotype and plasma analyte levels; 2) blood samples were collected at varying times (median 32 days) after the index event in order to avoid major influences from the acute event on plasma analytes, while this variable may have affected levels of these analytes, adjustment for time to sampling was included in statistical analysis in an effort to mitigate this; 3) CVs for the biomarkers sFlt-1 and sKDR data were high, without the achievement of improved assay performance this limits the accuracy and utility of these biomarkers 4) the analyses for VEGFR’s and pterins have only been conducted on a minority of the total CDCS cohort; 5) rigorous correction for multiple testing would adjust some of the uncorrected *p*-values across the *p* > 0.05 boundary; 6) the majority of the CDCS cohort are patients of European ancestry and the results should not be extrapolated to other populations; 7) the CDCS cohort was recruited over 9 years ago and therefore limited availability of recent treatment regimes such as dual anti-platelet therapy (received by 54% of the CDCS cohort) may have affected clinical outcome endpoints.

In summary we report an independent association of both plasma sFlt-1 and total pterin levels with all-cause and cardiovascular mortality in a cohort of patients with coronary artery disease followed up for several years after an index acute coronary event. These findings should be regarded as hypothesis generating and should be subject to validation studies in other cohorts.

## Conclusions

sFlt-1 and pterins appear to have potential as prognostic biomarkers in acute coronary syndromes patients. Genetic markers from VEGF system genes, particularly rs1870377, warrant further investigation as markers of levels of VEGF system components in these patients.

## Additional files

Additional file 1: **Table S1.** Baseline characteristics of the CDCS cohort stratified on whether they were assayed for sFlt-1 or not. **Table S2.** Genetic associations with VEGFR levels in baseline plasma and angiogram measurements from a) patients of European ethnic group and b) non-European ethnic group. **Table S3.** Baseline characteristics of the CDCS cohort stratified on whether they were assayed for neopterin or not. (PDF 112 kb)

Additional file 2: **Figure S1.** Receiver-operator curve analysis of NT-proBNP and sFlt-1 as predictors of mortality at 5 years of follow-up in the CDCS cohort. A receiver-operator curve analysis comparing the analytes NT-proBNP and sFlt-1 as predictors of mortality at 5 years of follow-up in the CDCS cohort. (PDF 76 kb)

## Abbreviations

7,8 NP: 7,8 dihydroneopterin
BNP: B-type natriuretic peptide
CAD: Coronary Artery Disease
CDCS: Coronary Disease Cohort Study
CHD: Coronary Heart Disease
CVD: Cardiovascular disease
Flt-1: fms related tyrosine kinase 1
HIF-1: Hypoxia Inducible Factor-1
HPLC: High performance liquid chromatography
KDR: Kinase insert domain receptor
nM: Nanomolar
NT-proBNP: N-terminal pro-B-type natriuretic peptide
sFlt-1: soluble FMS related tyrosine kinase 1
sKDR: soluble kinase insert domain receptor
SNP: Single nucleotide polymorphism
VEGF: Vascular endothelial growth factor
VEGFR1: Vascular endothelial growth factor receptor 1
VEGFR2: Vascular endothelial growth factor receptor 2
VEGFR3: Vascular endothelial growth factor receptor 3
μM: Micromolar
