# Supplementary material for: Plasma levels of soluble VEGF receptor isoforms, circulating pterins and VEGF system SNPs as prognostic biomarkers in patients with acute coronary syndromes
Source: BMC Cardiovasc Disord. 2018 Aug 15;18:169. doi: 10.1186/s12872-018-0894-1 (PMC6094571; doi:10.1186/s12872-018-0894-1)
Supplement: Supplementary file 1 — Table S1. Baseline characteristics of the CDCS cohort stratified on whether they were assayed for sFlt-1 or not. Table S2. Genetic associations with VEGFR levels in baseline plasma and angiogram measurements from a) patients of European ethnic group and b) non-European ethnic group. Table S3. Baseline characteristics of the CDCS cohort stratified on whether they were assayed for neopterin or not. (PDF 112 kb) [file 12872_2018_894_MOESM1_ESM.pdf]

Supplementary Table 1.

Baseline characteristics of the CDCS cohort stratified on whether they were assayed for sFlt-1 or not.

Baseline characteristics	**n**	**Mean ± SE or n (%)**	**n**	**Mean ± SE or n (%)**	**p-value**
	**Assayed for SFlt-1**		**Not assayed for sFlt-1**		
Male Gender	513	362 (70.6)	1554	1121 (72.1)	0.660
Age at baseline (years)	513	68.8 ± 0.53	1554	65.8±0.31	<0.001
Ethnicity (European, Maori & Pasifika, Other, Unknown)	513	Euro 447(87.1)<br>Maori/Pasifika 26(5.0)<br>Other 24 (4.6)<br>Unknown 16 (3.2)	1554	Euro 1340 (86.2)<br>Maori/Pasifika 76 (4.8)<br>Other 38 (2.4)<br>Unknown 100 (7.3)	0.493
Discharge Medications					
ACE inhibitor	513	301 (58.6)	1554	878 (56.5)	0.389
β-blocker	513	443 (86.3)	1554	1335 (85.9)	0.843
Diuretic	513	142 (27.6)	1554	424 (27.3)	0.888
Statin	513	451 (87.9)	1554	1375 (88.5)	0.689

Supplementary Table 2.

Genetic associations with VEGFR levels in baseline plasma and angiogram measurements from a) patients of European ethnic group and b) non-European ethnic group.

a) European ethnic group n=1747

i) ***VEGFR2*** **- rs1870377**

	n	TT	n	TA	n	AA	***p***
Age (years)	1000	67.5±0.38	643	68.3±0.46	104	67.9±1.02	0.395
Male Gender	1000	721 (72.1%)	643	449 (69.8%)	104	80 (76.9%)	0.278
sFlt-1 (pg/ml)*	264	107(101-114)	172	104(98.2-111)	25	129(103-161)	0.046
sKDR (pg/ml)	116	11100±236	71	10700±331	14	9350±470	0.041
Brandt Score	528	3.30±0.13	332	3.36±0.18	56	4.14±0.50	0.248

ii) ***VEGFR1*** **- rs748252**

	n	CC	n	CT	n	TT	***p***
Age (years)	710	67.9±0.44	790	67.9±0.43	235	67.5±0.80	0.912
Male Gender	710	520 (73.2%)	790	556 (70.4%)	235	164 (69.8%)	0.391
sFlt-1 (pg/ml) *	215	110(104-118)	201	104(97.9-110)	44	108(91.4-128)	0.293
sKDR (pg/ml)	99	11100±282	84	10700±257	18	10400±634	0.285
Brandt Score	389	3.21±0.15	404	3.41±0.16	119	3.72±0.32	0.065

b) non-European ethnic group n=264

i) ***VEGFR2*** **- rs1870377**

	n	TT	n	TA	n	AA	***p***
Age (years)	133	58.7±1.09	103	59.1±1.10	32	61.4±1.95	0.515
Male Gender	133	98 (73.7%)	103	72(69.9%)	32	26 (81.3%)	0.440
sFlt-1 (pg/ml)*	20	89.5(76.3-105)	16	98.4(80.0-121)	2	152(8.36-2760)	0.207
sKDR (pg/ml)	5	9410±934	4	11800±1360	1	7930	0.531
Brandt Score	50	2.97±0.48	45	3.60±0.48	13	2.77±0.92	0.507

ii) ***VEGFR1* - rs748252**

	n	CC	n	CT	n	TT	***p***
Age (years)	91	59.3±1.36	131	59.1±0.99	42	58.5±1.74	0.929
Male Gender	91	70 (76.9%)	131	94 (71.8%)	42	29 (69.0%)	0.563
sFlt-1 (pg/ml)*	9	108(74.5-156)	23	93.9(80.3-110)	6	86.7(70.7-106)	0.563
sKDR (pg/ml)	2	9400±1130	6	10700±1260	2	9510±1400	0.924
Brandt Score	38	2.90±0.55	53	2.95±0.40	16	4.91±1.00	0.083

*geometric mean

Supplementary Table 3.

Baseline characteristics of the CDCS cohort stratified on whether they were assayed for neopterin or not.

Baseline characteristics	**n**	**Mean ± SE or n (%)**	**n**	**Mean ± SE or n (%)**	**p-value**
	Assayed for neopterin		Not assayed for neopterin		
Male Gender	142	97 (68.3)	1925	1382 (71.8)	0.377
Age at baseline (years)	142	66.9 ± 1.00	1925	66.46±0.28	0.667
Ethnicity (European, Maori & Pasifika, Other, Unknown)	142	Euro 133(93.6)<br>Maori/Pasifika 6 (4.3)<br>Other 1 (0.7)<br>Unknown 2(1.4)	1925	Euro 1657 (86.1)<br>Maori/Pasifika 127 (6.8)<br>Other 77 (4.0)<br>Unknown 66 (3.3)	0.070
Discharge Medications					
ACE inhibitor	142	83 (58.5)	1925	1103 (57.3)	0.781
β-blocker	142	126 (88.7)	1925	1648 (85.6)	0.306
Diuretic	142	44 (31.0)	1925	522 (27.1)	0.313
Statin	142	126 (88.7)	1925	1700 (88.3)	0.874
