# Supplementary material for: Plasma levels of soluble VEGF receptor isoforms, circulating pterins and VEGF system SNPs as prognostic biomarkers in patients with acute coronary syndromes
Source: BMC Cardiovasc Disord. 2018 Aug 15;18:169. doi: 10.1186/s12872-018-0894-1 (PMC6094571; doi:10.1186/s12872-018-0894-1)
Supplement: Supplementary file 2 — Figure S1. Receiver-operator curve analysis of NT-proBNP and sFlt-1 as predictors of mortality at 5 years of follow-up in the CDCS cohort. A receiver-operator curve analysis comparing the analytes NT-proBNP and sFlt-1 as predictors of mortality at 5 years of follow-up in the CDCS cohort. (PDF 76 kb) [file 12872_2018_894_MOESM2_ESM.pdf]

Supplementary Figure 1.
Receiver-operator curve analysis of NT-proBNP and sFlt-1 as predictors of mortality at 5 years of follow-up in the CDCS cohort.

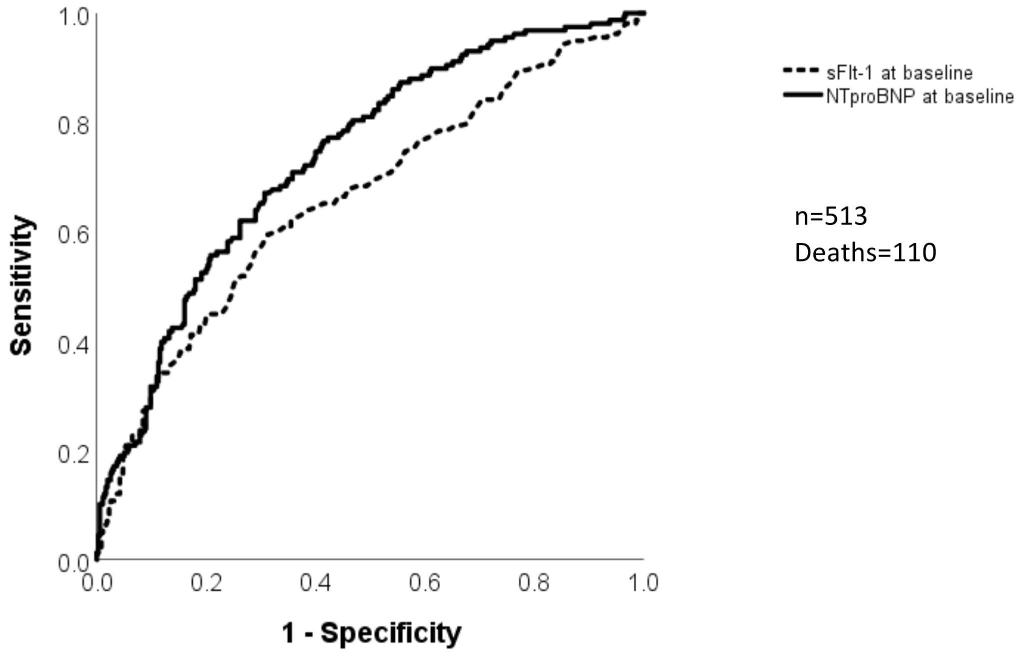

Analyte	AUC	95% CI for AUC	p-value
sFlt-1 at baseline	0.633	0.571-0.695	<0.001
NTproBNP at baseline	0.767	0.719-0.815	<0.001
